# Supplementary material for: Haplotype-Resolved DNA Methylation at the APOE Locus identifies Allele-Specific Epigenetic Signatures Relevant to Alzheimer’s Disease Risk
Source: bioRxiv. 2025 Jul 2:2025.07.01.662592. Preprint. [Version 1] doi: 10.1101/2025.07.01.662592 (PMC12236645; doi:10.1101/2025.07.01.662592)
Supplement: Supplement 2 [file NIHPP2025.07.01.662592v1-supplement-2.pdf]

bioRxiv preprint doi: https://doi.org/10.1101/2025.07.01.662592; this version posted July 2, 2025. The copyright holder for this preprint (which was not certified by peer review) is the author/funder. This article is a US Government work. It is not subject to copyright under 17 USC 105 and is also made available for use under a CC0 license.

Supplementary Figures:

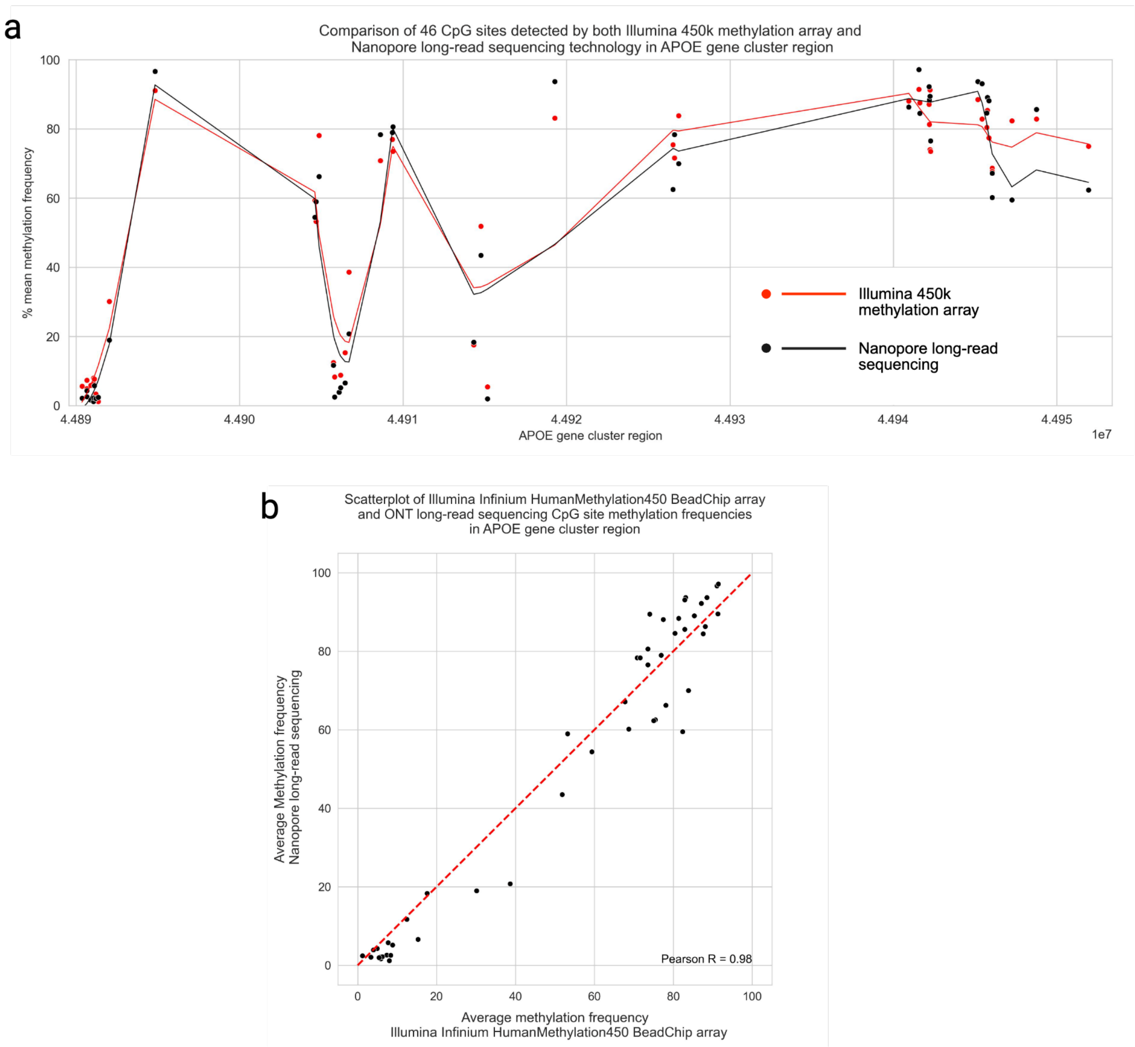

Supplementary Figure 1. Comparison of the 46 CpG sites detected by both platforms. a) Mean methylation frequencies of the 46 CpG sites detected in two separate EUR-ancestry brain tissue cohorts sequenced by the Illumina Infinium HumanMethylation450 BeadChip array (red) and the ONT long-read sequencing technology (black) within the *APOE* cluster region (*TOMM40, APOE, APOC1,* and *APOC4-APOC2* genes; hg38 coordinates chr19:44889556-44953378). **b)** Scatter plot of the Illumina Infinium HumanMethylation450 BeadChip array and the long-read sequenced CpG site methylation frequencies in the *APOE* cluster region. Pearson R = 0.98, P-value = 3.506e-33.

bioRxiv preprint doi: https://doi.org/10.1101/2025.07.01.662592; this version posted July 2, 2025. The copyright holder for this preprint (which was not certified by peer review) is the author/funder. This article is a US Government work. It is not subject to copyright under 17 USC 105 and is also made available for use under a CC0 license.

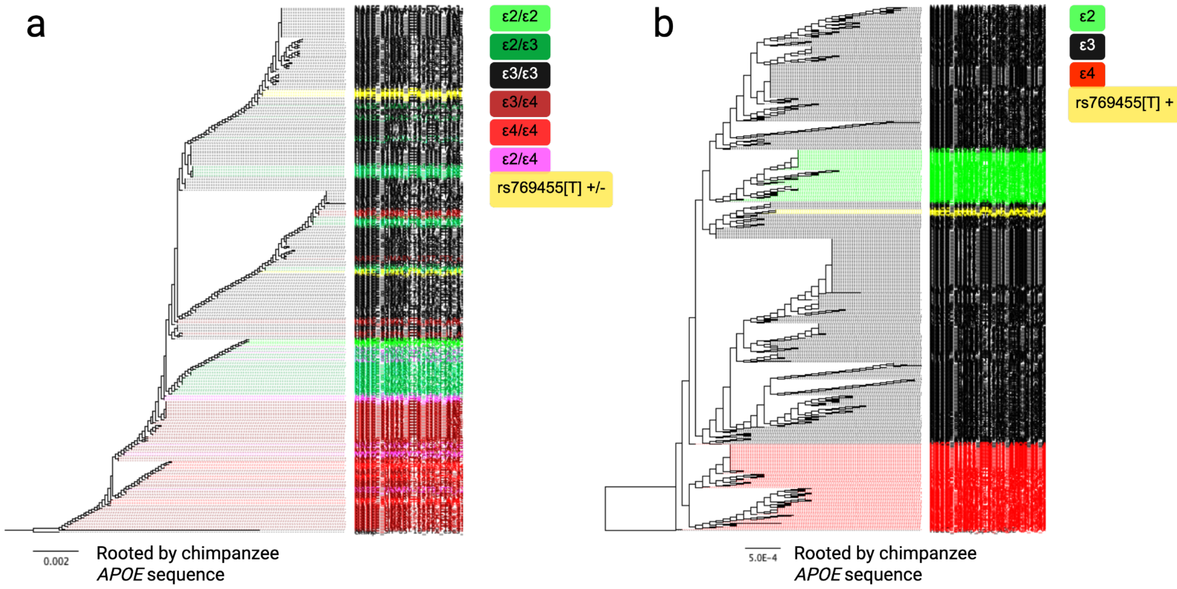

Supplementary Figure 2. Dendrograms depictions of *APOE* allele separation at the allele and genotype levels. a) Dendrograms of the *APOE* gene region (chr19:44905791-44909393, hg38) generated from 332 unphased genotypes from the NABEC and HBCC cohorts. Branches are colored by *APOE* genotype. Only basecalls with >20× coverage were included. **b)** Dendrogram of the *APOE* region generated from 664 phased haplotypes from the NABEC and HBCC cohorts. Branches are colored by allele type: *APOE*-ε2 in green, *APOE*-ε4 in red, and rs769455[T] in yellow. Only positions with > 10× coverage were included. In both panels, each branch represents the aligned consensus FASTA sequence for an individual *APOE* genotype (a) or haplotype (b). Dendrograms were rooted using the ancestral chimpanzee *APOE* sequence. See Methods for full details on dendrogram generation.

bioRxiv preprint doi: https://doi.org/10.1101/2025.07.01.662592; this version posted July 2, 2025. The copyright holder for this preprint (which was not certified by peer review) is the author/funder. This article is a US Government work. It is not subject to copyright under 17 USC 105 and is also made available for use under a CC0 license.

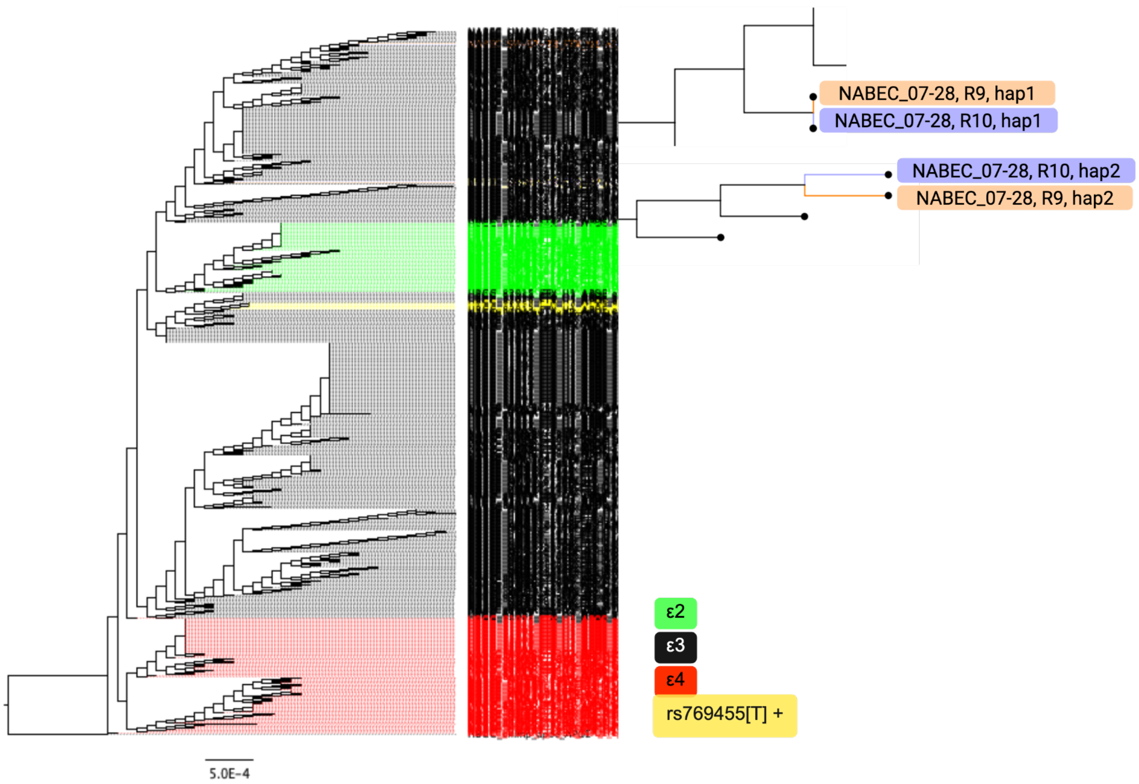

Supplementary Figure 3. Benchmarking the effect of Nanopore flow cell chemistry on phased allele separation in a dendrogram. A copy of the dendrogram featured Supplementary Figure 2 that has been expanded to show one NABEC sample (NABEC_07-28) that was sequenced with both R9 (orange) and R10 (purple) chemistries. The branches containing each sample haplotype have been magnified on the right side of the dendrogram.

bioRxiv preprint doi: https://doi.org/10.1101/2025.07.01.662592; this version posted July 2, 2025. The copyright holder for this preprint (which was not certified by peer review) is the author/funder. This article is a US Government work. It is not subject to copyright under 17 USC 105 and is also made available for use under a CC0 license.

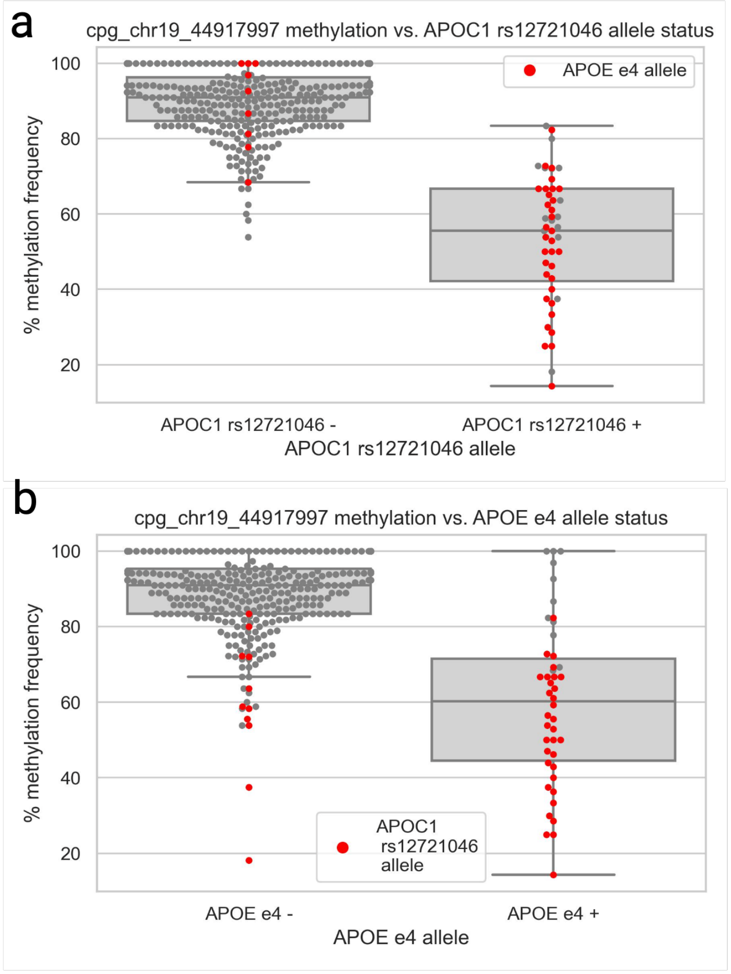

Supplementary Figure 4. Assessing *APOE* e4 and *APOC1* rs12721046[A] allele effects on methylation frequency differences at NABEC CpG site cpg_chr19_44917997. a) A box-and-whisker plot showing the methylation frequency difference at NABEC CpG site cpg_chr19_44917997 when stratified by the *APOC1* rs12721046[A] allele (with the *APOE*-ε4 alleles highlighted in red and included as a covariate in the linear regression analysis). **b)** A box-and-whisker plot showing the methylation frequency difference at NABEC CpG site cpg_chr19_44917997 when stratified by the *APOE*-ε4 allele (with the *APOC1* rs12721046[A] + alleles highlighted in red and included as covariate in linear regression analysis). The dots on the left half of the box plots depict sample haplotypes that do not have the allele of interest (denoted by "-") and the right half show samples that do have the allele of interest (denoted by "+").

bioRxiv preprint doi: https://doi.org/10.1101/2025.07.01.662592; this version posted July 2, 2025. The copyright holder for this preprint (which was not certified by peer review) is the author/funder. This article is a US Government work. It is not subject to copyright under 17 USC 105 and is also made available for use under a CC0 license.

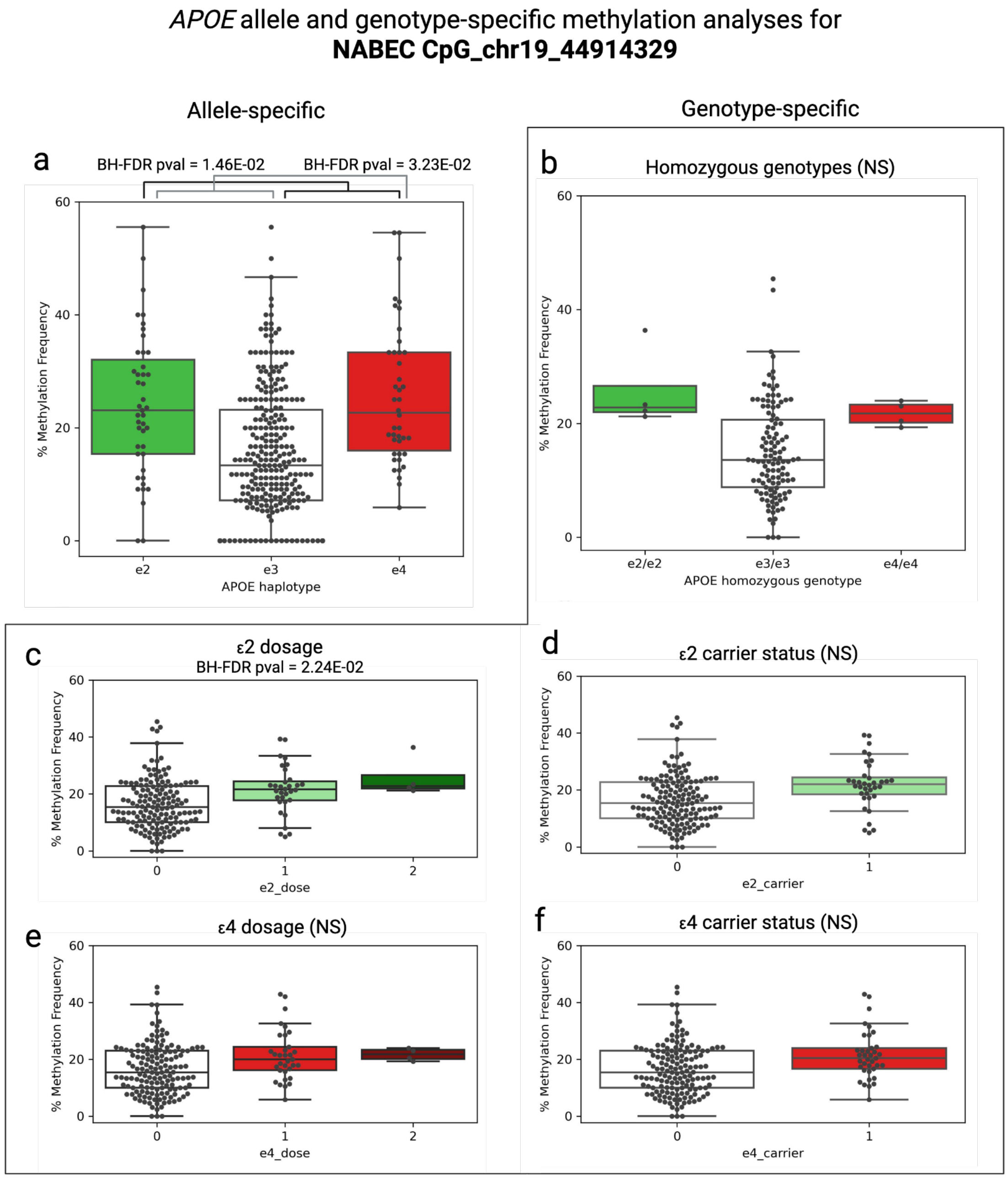

Supplementary Figure 5. Box-and-whisker plots of the ε4 *APOE* allele-specific methylation frequencies for NABEC CpG site cpg_chr19_44914329 as determined by allele-specific and genotype-level methylation analyses. a) The boxplot of allele-specific methylation analysis results shown in Figure 3b. **b-f)** Genotype-level methylation analyses based on homozygous genotypes (b), ε2 dosage and carrier status (c, d) and ε4 dosage and carrier status (e, f). NS = BH-FDR corrected p value was not significant.

bioRxiv preprint doi: https://doi.org/10.1101/2025.07.01.662592; this version posted July 2, 2025. The copyright holder for this preprint (which was not certified by peer review) is the author/funder. This article is a US Government work. It is not subject to copyright under 17 USC 105 and is also made available for use under a CC0 license.

Supplementary Tables:

Table S1. Summary table of previous studies of *APOE* methylation in brain and blood tissue samples including the number and type of samples analyzed, methylation sequencing technique used, number of CpG sites identified and where they were located in the genome.

bioRxiv preprint doi: https://doi.org/10.1101/2025.07.01.662592; this version posted July 2, 2025. The copyright holder for this preprint (which was not certified by peer review) is the author/funder. This article is a US Government work. It is not subject to copyright under 17 USC 105 and is also made available for use under a CC0 license.

Table S2: Summary of cohort demographics including the total number samples, number of male and female samples, mean age, age range, ancestry, diagnosis, sample type, sequencing coverage and sequencing N50.

Table S3: Haplotype-specific sequencing statistics for each NABEC and HBCC sample used in the study including the mean and median sequencing lengths, N50, number of reads, and mean coverage.

Table S4: Significant allele-specific differentially methylated CpG sites analyzed using genotype-level methylation analysis methods.

Table S5: Linear regression results of *APOE* cluster region gene expression associated with *APOE* alleles.
